# Formulation, development and evaluation of bifunctionalized nanoliposomes containing *Trifolium resupinatum* sprout methanolic extract: as effective natural antioxidants on the oxidative stability of soybean oil

**DOI:** 10.1186/s13065-019-0594-7

**Published:** 2019-06-28

**Authors:** S. Zahra Sayyed-Alangi, Meysam Nematzadeh

**Affiliations:** 1 Department of Chemistry, Azadshahr Branch, Islamic Azad University, Azadshahr, Iran; 2 Department of Food Engineering, Azadshahr Branch, Islamic Azad University, Azadshahr, Iran

**Keywords:** Antioxidant, *Persian* clover sprout, *Trifolium resupinatum*, Nanoliposome

## Abstract

**Background:**

The various extracts of *Trifolium resupinatum* (*Persian* clover) sprout was obtained by using different solvents and microwave assisted extraction in the present study. Then, the bifunctionalized nanoliposomes were prepared and added to soybean oil for evaluating their effect on deferring the oxidation process.

**Methods:**

The total phenol and antioxidant activity of the extracts was determined by using the free radical scavenging assay. Then, various nanoliposomal structures of the methanolic extract of *Persian* clover sprout (PCSE) were prepared by using six several formulations containing different ratios of soybean oil, lecithin and the extract. Afterward, the most stable nanoliposome was bifunctionalized by using WPC and pectin (PCSEN-W and PCSEN-WP, respectively). The size and zeta potential of nanoparticles were measured. Furthermore, in order to evaluate the effects of PCSE, PCSEN, PCSEN-W and PCSEN-WP at 100–300 ppm concentrations in deferring the oxidation process of soybean oil, the heat treatment tests were applied (PV and TBA) at 63 °C within a 20-day period.

**Results:**

The methanolic extract had the highest level of total phenol and antioxidant activity. The results of creaming index and microencapsulation efficiency were exhibited that formulation containing 30% oil, 5% lecithin and 2% the extract was led to the production of the most stable nanoliposomal structure (PCSEN). The size of nanoparticles was in the range of 282.5–491.2 nm. Zeta potential of the samples was obtained in the range between − 56.9 and − 36.3 mV. Polydispersity index of them was ranged from 0.424 to 0.541. The results were confirmed the existence of stable nanoliposomal systems. The results of the PV and TBA values of the extracts in free and nanoliposomal forms were shown that the nanoliposomal forms had very good antioxidant activity against the oxidation process in soybean oil.
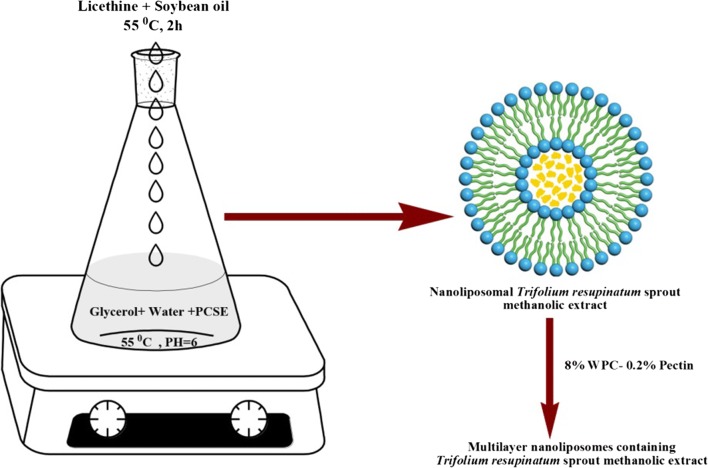

## Introduction

Clovers have been considered as one of the most widely used medicinal plants in traditional medicine in the past. Even today, it has a position far higher than the past. For example, the scientific study performed on red clover in order to prove its anticarcinogens properties [1]. Furthermore, the potential anticancer of some clovers confirms [2–4]. Also, some clovers are known as traditional wild food [5]. Clover sprout is one the nature miracles in the treatment of anemia and low platelet count, certainly, it should also be noted that healing properties of this plant are unique in its kind and perhaps these healing properties can be found in expensive dietary supplements. Scientific study on anticancer properties of clovers is still new. But, it proves red clover’s future cancer treatment role [2–4]. Also, clover sprouts contain digestive and antioxidant enzymes.

Synthetic antioxidants are widely used in various industries, but toxic and cancer-causing compounds resulting from the decomposition of these materials during processing of products has caused a great concern regarding this type of additives. So, the need for potent antioxidants with less toxicity and more effectiveness is an inevitable necessity. Nowadays, many nutritionists recommend using plants, fruits and vegetables in order to provide the antioxidants needed by the body, since usually consumption of herbal antioxidants makes fewer side effects and provides better treatment [6]. Natural antioxidants are usually expensive, despite their high nutritional value and healing properties so it is very important to find a way to produce cheap natural antioxidants and yet with the same nutritional value and healing properties. One of the problems in using extracts and essences as natural antioxidants is their instability under environmental conditions, adverse and astringent taste for use in various food and medicine [7].

Encapsulation is a technique in which solids, liquids or gaseous substances within small capsules are encapsulated, packaged and released under certain conditions [8]. This technique has been used since 60 years ago and so far growing advancements has been done in this area [9, 10]. The purpose of encapsulation can be explained as protecting core materials against decomposition, decreased activity by exposure to the surrounding environment, reduction of evaporation and transfer of core material to the external environment, modifying the physical conditions of the core material and thereby making more favorable conditions for transportation and industrial applications and covering undesirable flavor and aroma of some core materials [9, 11].

Liposomes are little round vesicles which can be made from cholesterol, natural nontoxic phospholipids, glycolipids or sialic acid [12]. Study on liposome technology has advanced from conventional vesicles to ‘second-generation liposomes’, in which long-circulating liposomes are achieved through modifying the lipid composition, size, and charge of the vesicle. They are a very effective tool in different sciences, including mathematics and theoretical physics, biophysics, chemistry, colloid science, biochemistry and drug delivery systems. In this regard, nanoliposome applications for medical uses extend about 50 and 500 nm as nanomedical gadgets [13].

Antioxidant activity, total phenolic and flavonoid content of different solvent extracts from in vivo and in vitro grown *Trifolium pratense* L. (red clover) investigated [14]. Also, Mirzaee and Dehghan [15] evaluated the phenolic content and antioxidant activities of *Trifolium subterraneum*. But, there is no study about preparation, formulation and characterization of encapsulated *Persian* clover sprout (PCS) extract in nanoliposomal structures, and also their properties as antioxidant. This study has evaluated various formulations to provide the most stable functionalized nanoliposomes of PCS methanolic extract (PCSE). Then, their antioxidant properties have investigated against the oxidation process in soybean oil.

## Methods

### Materials

PCS (*Trifolium resupinatum*) was bought from nearby market in Gorgon, Golestan, Iran. Unadulterated soybean oil was acquired from Alia Golestan Company (Kordkooy, Iran). Folin-Ciocalteo reagent, trichloroacetic acid, iron (III) chloride, sodium phosphate, sulphuric acid, ammonium molybdate, potassium iodide, acetic acid, sodium thiosulfate, butanol, thiobarbituric acid and whey protein concentrate (%80 protein) with analytical grade were prepared by Merck Company. Gallic acid, sodium carbonate, 2,2-diphenyl-1-picrylhydrazyl, acetone, methanol and ethanol were purchased from Sigma Company. Sodium azide was obtained from Sigma-Aldrich. Citrus pectin (GA: > 65%) was purchased from MP biomedical (Netherland).

### Plant identification

The sample of *Trifolium resupinatum* seed was bought from the Gorgan region of Golestan province (Iran). Then, they were transferred to the Gonbad Kavous University Herbaryum center (GKUH) for identification. Identification of the sample was done by referring to Feedipedia [16]. Also, they are stored at the herbaryum center with code number of 803582-GKUH.

### Sample preparation

PCS was cleaned and dried in an oven at 45 °C, and then it was changed over into powder by using a mill and sieved through the mesh 40. After that, it was stocked in the air and moisture resistant packages and kept on − 18 °C for further examinations.

### Microwave assisted extraction of PCS

The 5 g of the dried and powder PCS was blended with 50 ml of the various solvents (80% methanol, 80% ethanol, 80% acetone and water) in a 250 ml volumetric flask. The mixtures were kept at room temperature for 8 h. In this regard, the extraction was performed during 15 min with magnetic stirring (8 s power on and 15 s power off in order to avoid super-boiling of solvent) with a 900 W microwave power. Then, the extract was filtered and a rotary evaporator (Buchi-V-800, Switzerland) was applied to remove the solvent at 50 °C under decreased pressure. In the end, a freeze-drier (Operon FDB-5503) was used to dry the produced extract at − 20 °C. The resulted extracts were stored in the freezer at − 18 °C for further testing [17].

### Measurement of the total phenol and free radical scavenging ability

The Folin–Ciocalteu method was applied to determine the phenolic compounds [18, 19]. The free radical scavenging potential of the samples was also evaluated by the method of Shimada et al. [20].

### Biopolymer solution preparation

Material solutions as the outer wall phase of bifunctionlized nanoliposome were formulated by pectin and whey protein concentrate (WPC). The powdered pectin was dissolved in distilled water and stirred at 50 °C for 30 min. Afterward, the mixture was retained at room temperature overnight. In the case of WPC, its solution was preserved refrigerated for 24 h to complete hydration. After that, the pH of the solution was controlled with phosphate buffer (pH = 6). Then, the solution was heated at 70 °C for 20 min, and cooled rapidly. Finally, the solution of WPC–pectin was got ready by adding pectin solution within the solution of WPC, and stirring at room temperature for 1 h. Afterward, the solution was regulated at pH 6 by using phosphate buffer. It was maintained in a refrigerator overnight. 0.004% sodium azide was existed in the solutions as an antimicrobial element [21].

### Various designs for formulation optimization of the nanoliposomes containing PCSE

In order to produce the optimized nanoliposome, different formulations were designed for producing nanoliposomes (PCSEN) prepared by the Mozafari method based on the heating technique [21] with some few changes, which they are shown in Table 1.

**Table 1:** The composition of the designed formulations for nanoliposomes containing PCSE, lecithin and soybean oil

Formulation	1	2	3	4	5	6
Extract (%)	2	4	6	2	4	6
Lecithin (%)	5	5	5	5	5	5
Soybean oil (%)	30	30	30	35	35	35

The concentration of all the materials used in the formula calculated by w/w

The production process of nanoliposomes was done as follows:

Firstly, an organic phase was generated with the incorporation of lecithin into soybean oil. This mixture was heat-treated in a water bath at 55 °C temperature for 2 h. 3% glycerol was combined with 7 ml distilled water, and then PCSE was dissolved in hot water at 40 °C temperature as an aqueous phase. In the following step, the solution of water and 3% glycerol was combined to the extract at 55 °C temperature and pH = 6 while mixing (approx. 500 rpm) on a hotplate stirrer (HCR2 Gerhardt Germany). Afterwards, drop wise of organic phase was added to the produced aqueous phase that led to the preparation of the emulsions. For producing nanoliposomes, the prepared emulsions were homogenized (8000 rpm for 8 min) by a rotor-stator homogenizer (IKA ultra Turrax, Germany).

### Preparation of PCSE-loaded functionalized nanoliposome

After choosing the most optimized formulation of the prepared nanoliposomes, 8% WPC was used to produce monofunctional nanoliposome (PCSEN-W) for its coverage. Also, 0.2% pectin–8% WPC with a proportion of 30% initial nanoliposome and 70% combination of pectin and WPC (1:1), respectively, was used for producing bifunctional nanoliposome (PCSEN-WP) [22].

### Assessment of creaming index

Determination of stability was performed through measuring the creaming index by using the method of Nikiforidis and Kiosseoglou [23]. The 20 ml of the emulsion was placed in a glass vessel at room temperature for 24 h. Then, the creaming index was estimated according to the formula (Eq. 1):

1 $${\text{CI}}( \%) = \frac{\text{HS}}{{\text{HE}}} \times 100$$

where CI is creaming index, HS is the height of the serum layer and HE is the total height of the emulsion.

### Measurement of encapsulation efficiency

Encapsulation efficiency of nanoliposomes containing PCSE was done by using the method of Mohammadi et al. [24]. In order to evaluate the encapsulation efficiency percentage, 10 ml of the encapsulated clovers formulation was placed in a refrigerated centrifuge at 4 °C for 15 min at 45,000 rpm. Then, the surface solution was separated and dissolved in 0.1% normal chloroform solution until the liposomes were broken. Afterwards, the transparence extract was filtered and extracted with 0.45 mm membrane. After that, the resulting solution was poured into the cuvette and it was identified by the optical absorption spectrophotometer at 240–250 nm.

### Evaluation of droplet size, polydispersity and zeta potential of bifunctional nanoliposomes

The z-average droplet size, particle size distribution (PSD), polydispersity index (PdI) and zeta potential of the nanoliposomes (PCSEN, PCSEN-W and PCSE-WP) were assessed using a commercial Dynamic Light Scattering instrument (Zetasizer Nano ZS; Malvern Instruments, Malvern, UK) at 25 °C and pH = 7.4. The instrument was equipped with a He–Ne laser source (λ = 633 nm) and operated at an angle of 173°. The suspension was generated through scattering the nanoliposomes in deionized water with a proportion of 1:5 [25].

### Evaluation of oxidative stability of the nanoliposomes on soybean oil

In order to evaluate the antioxidant activity of the samples in oil, deodorized and bleached soybean oil with no additives was prepared. PCSE, PCSEN, PCSEN-W and PCSEN-WP in three levels (100, 200 and 300 ppm), and the synthetic antioxidant (BHT) at 100 and 200 ppm concentrations were added to oil and mixed by a magnetic heater (Fater Electronics) in 30 min. Soybean oil with no additives was also selected as a control sample. The samples were transferred into a series of glass and placed in an oven at 63 °C for 20 days.

### Determination of peroxide value (PV) and thiobarbituric acid (TBA) values

Peroxide value (PV) and thiobarbituric acid (TBA) were measured in 4-days intervals up to 20 days and they were determined according to AOAC [26] method.

### Statistical analysis

Design-expert 8.0.4 Trial software (6th version, 2000) was used to determine the best treatment and Image J software was applied to choose the best formula in term of the size of the nanoliposomes. This study was conducted in a completely randomized design and the obtained results were analyzed by using analysis of variance (ANOVA). Mean comparison was done by using Duncan’s multiple range test at the 5% possibility level (*p *< 0.05). Data were analyzed by using IBM SPSS Statistics V22.0 software.

## Results and discussion

### Evaluation of the total phenol content of various extracts from PCS

**Fig. 1:**
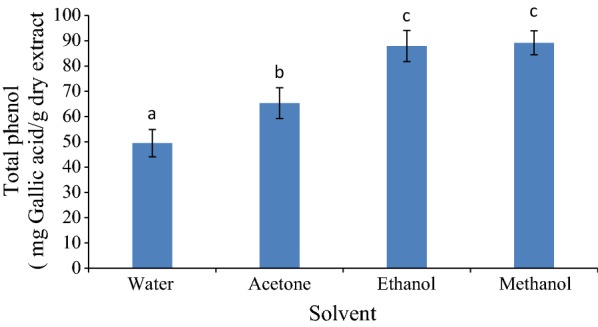
The total phenolic content of various extracts from PCS (mg Gallic acid/g dry extract). Different letters indicate significant differences (*p *< 0.05)

The results related to the total amount of phenolic compounds in various extracts of PCS have been shown in Fig. 1. The results showed that there was a significant difference between the total phenolic content in the different extracts (*p *< 0.05). Among the extracts, the methanolic extract with 89.18 ± 4.73 mg Gallic acid/g dry matter and the water extract with 49.5 ± 5.409 mg Gallic acid/g dry matter sample of extract had the highest and lowest contents of phenolic compounds, respectively. Various solvents have different capabilities on the extraction of herbal phenolic compounds. Water is known as the cheapest and most available solvent for extraction of herbal compounds; however, it shows a small level of ability to extract effective herbal compounds since these compounds are mainly non-polar and fat-soluble. Methanol can be more effective in extracting organic polar compounds due to its organic polarization nature. Therefore, the results of phenolic content indicated that the compounds in PCS were mostly organic polar compounds. Similar results reported by Debnath et al. [27]. These researchers prepared the alcoholic extracts of *Gardenia* plant by the maceration method for 24 h at room temperature and water extract at a temperature of 85 °C for 3 h. The total phenolic contents in water and alcohol extracts were 44.8 and 53.5 mg/g, respectively. Also, Mirzaee and Dehghan [15] found similar results so that the methanolic extract of clover has a high level of phenolic content. Moreover, Khorasani Esmaeili et al. [14] in investigation on red clover were found that the methanolic extract had the most phenolic contents.

### Assessment of DPPH free radical scavenging power of different extracts of clover sprout

The results of DPPH free radical scavenging power in different extracts of PCS are shown in Table 2. As it can be seen, there is a significant difference between all concentrations of the extract as well as between the various extracts at a similar concentration (*p *< 0.05). Type and concentration of the extracts were the effective parameters in the free radical control. The scavenging ability of DPPH radicals enhanced by increasing the concentration of each extract. According to the results, it was shown that free radical scavenging capability of alcoholic extracts, especially the methanolic extract, was higher than other extracts (Table 2). So that, the highest total phenol content was belonged to the methanolic extract at 1000 ppm concentration (71.88 ± 0.95%). The lowest free radical scavenging power was obtained in the water extract at 100 ppm concentration (5.37 ± 1.75%). These results were confirmed that there was a direct relationship between the phenol contents and the potential of radical inhibitory release.

**Table 2:** DPPH free radical scavenging activity (%) in the various extracts of PCS

Concentration (ppm)	Extract			
	Water	Acetone	Ethanol	Methanol
100	5.37 ± 1.75^Ea^	16.12 ± 1.24^Eb^	21.24 ± 2.10^Ec^	24.98 ± 1.52^Ed^
250	7.20 ± 2.21 ^Da^	20.25 ± 1.38^Db^	31.45 ± 1.04^Dc^	37.55 ± 1.30^Dd^
500	9.02 ± 1.48^Ca^	27.79 ± 2.03^Cb^	42.75 ± 2.18^Cc^	49.58 ± 0.95^Cd^
750	11.26 ± 2.86^Ba^	32.51 ± 0.31^Bb^	52.78 ± 1.25^Bc^	63.43 ± 0.58^Bd^
1000	13.09 ± 2.10^Aa^	41.17 ± 2.15^Ab^	57.02 ± 1.76^Ac^	71.88 ± 0.95^Ad^

Different capital letters indicate significant difference in each column (*p *< 0.05)

Different small letters indicate significant difference in each row (*p *< 0.05)

The presence of compounds with circular structure is the most important factor in the incidence of antioxidant activity in extracts, which are often insoluble or less soluble compounds in water. Whatever solvent extraction capability for these compounds is more the obtained extract will also show a higher antioxidant activity. Moreover, the simultaneous presence of antioxidant compounds in the extract leads to a synergistic effect, which in turn increases the antioxidant activity of the extract. Candan et al. [28] reported similar results after studying the antioxidant activity of water and alcoholic extracts of *Achillea millefolium* plant. They stated that the most extraction of compounds involved in antioxidant activity was done by methanol as a solvent. Khorasani Esmaeili et al. [14] in the study on red clover were obtained that the methanol extract had the most antioxidant activities.

### Identification of optimum nanoliposome containing PCSE

**Fig. 2:**
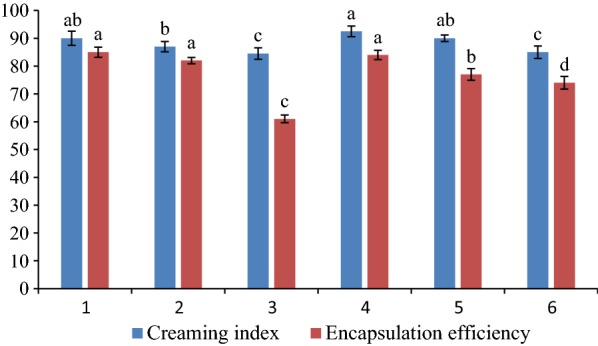
Creaming index and microencapsulation efficiency of various formula of initial nanoliposomes. Different small letters in columns of similar colors indicate significant differences (*p *< 0.05)

Since the methanolic extract has the highest amount of total phenol and free radical scavenging potential, it was used to provide various formulations of the initial nanoliposomes (Table 1). The results of creaming index and microencapsulation efficiency tests was used to select a stable nanoliposome formulation containing PCSE (Fig. 2).

Creaming index provides indirect data about the accumulated amount of droplets in the emulsion. Further accumulation is effective in increasing the size of the droplets, hence it increases the speed of creaming [29]. Nanoliposomes with a creaming index of close to 100 are nanoemulsion systems with more stability. The results of this test on various samples showed that there was a significant difference in the creaming index of various formulations (Fig. 2). Among the applied formulations, the sample 4 (35% oil, 5% lecithin and 2% extract) and formulation 3 (30% oil, 5% lecithin, and 6% extract) with the creaming index values of 92.5 ± 1.895 and 84.5 ± 2.05, respectively, had the highest and lowest levels of creaming index. As it can be seen, increasing concentration of the extract in various formulations of initial nanoliposome reduced system stability and consequently it decreased the creaming index (Fig. 2). Also, increasing the amount of the oil used in the formulations was led to a relative increase in creaming index and the stability of the system containing nanoliposomes. But this increase is not significant (Fig. 2). So, there was no significant difference between the samples 3 and 6 in the creaming index.

In general, any factor that weakens the hydrophilic and hydrophobic interactions in emulsion phases of nanoliposomes will lead to the system instability [30]. Increasing concentration of the extract, as an important factor in the weakening of these interactions, can lead to reduce the stability of the system containing nanoliposomes [30]. Xia et al. [31] indicated that the amount of oil used for preparing the formulation had the most important effect on the formation of nanoliposomes containing coenzyme Q10. So, the parameters related to sustainability of nanoliposomes could be altered by changing the amount of oil consumption, which is consistent with the results of this study.

The results of microencapsulation efficiency exhibited that there was a significant difference between the microencapsulation efficiency of different samples (Fig. 2). The samples 1 and 4 had the highest level of microencapsulation efficiency, which they were no significant difference together. The lowest level of it belonged to the sample 3 (Fig. 2). Increasing the amount of extract in the formulations caused to reduce the microencapsulation efficiency as well as creaming index. Microencapsulation efficiency is an important parameter in assessing the nano-carriers and it depends on factors such as the nature of the encapsulated material (lipophilic or hydrophilic), the nature and concentration of the used phospholipid, the type and concentration of stabilizer compounds, production method of liposome and environmental conditions such as pH and temperature [32]. Since increasing the hydrophilic parts in the formulation of encapsulated material is an important process in reducing capsulation efficiency, it can be concluded that encapsulated phase had been enhanced by increasing the amount of hydrophilic parts extract. Moreover, a decrease in proportion of the oil and lecithin phase to the extract was also an important factor in reducing the microencapsulation efficiency at high concentrations of the extract. Increased oil in the formulation was also caused a slight increase in the efficiency of formulation, which it was not significant (Fig. 2). PCSE has a range of compounds dissolved in fat and water phases by itself, which depended on their combination could be established in hydrophilic and hydrophobic parts of the liposomes. Mohammadi et al. [33] reported that highly hydrophobic nature of encapsulated matter was the reason for the high efficiency of the coverage. Sebaaly et al. [34] in the preparation and characterization of clove essential oil-loaded liposomes proved that the essential oil amount had a very important effect on the size and distribution of liposome particles. Their findings were in agreement with the results of the present study.

Given to evaluation of the stability of the produced nanoliposomal systems containing PCSE based on the results of creaming index and microencapsulation efficiency as well as the use of less oil in the formulation to produce a healthier and more economically safe product, the formulation 1 was generally used as the basis for producing functional nanoliposomes.

### Characterization of PCSE-loaded functionalized nanoliposomes

Nanoliposomes are very flexible and sensitive compounds to internal and external stresses, hence, researchers have turned to producing multifunctionalized nanoliposomes in order to resolve this shortcoming [35].

### Comparing nanoliposomes (PCSEN, PCSEN-W and PCSE-WP) in creaming index and microencapsulated efficiency

After selecting the formulation 1 (containing 30% the oil, 5% lecithin and 2% PCSE) as the base formulation, the mono and bifuntional nanoliposomes were produced by using the WPC and pectin (PCSEN-W and PCSE-WP, respectively). The results of comparing nanoliposomes (PCSEN, PCSEN-W and PCSE-WP) in creaming index and microencapsulated efficiency are reported in Table 3.

**Table 3:** Creaming index and microencapsulated efficiency of PCSEN, PCSEN-W and PCSE-WP

Treatment	Creaming index	Encapsulation efficiency
PCSEN	92 ± 1.50^b^	85 ± 1.67^b^
PCSEN-W	95 ± 1.10^a^	90 ± 1.13^a^
PCSE-WP	94 ± 1.70^ab^	91 ± 1.13^a^

Different small letters in each column indicate significant differences (*p *< 0.05)

The results demonstrated that the increased number of nanoliposome layers caused to raise the creaming index of treatments significantly. So, creaming index of the initial nanoliposome increased from 92 to 94 and 95 by adding layers of WPC and pectin (Table 3). In this method, whatever the amount of creaming index gets closer to 100, there will be a more stable emulsion. Also, this index can be used as a factor for indirect measuring the accumulated amount of nanoliposome particles in the emulsions [24, 33].

Frenzel and Steffen-Heins [36] during the production of multilayer liposomes by a whey protein isolate showed that the use of a protein coating improved the stability of the liposomes. Along with the results obtained in this study, Toniazzo et al. [37], during the preparation of multilayer liposomes containing beta-carotene, found that the presence of hydrophilic compounds such as xanthan and guar led to a significant increase of stability in the initial liposomes. As if, the size of multilayer liposomes did not change during the storage over a long period of time. Moreover, the beta-carotenes encapsulated in multilayer liposomes revealed their antioxidant properties for a longer period. Gibis et al. [38] used multilayer liposomes to microencapsulate the extract of *Hibiscus sabdariffa*. For this purpose, they applied layers of chitosan and pectin as the outer layers of liposome. Their finding exhibited that using the outer layers in preparing nanoliposome led to increase in the stability of the produced liposomes, which was consistent with the results obtained in this study.

The results showed that a significant increase in microencapsulation efficiency of treatments caused by a increased number of nanoliposome layers. So, the microencapsulated efficiency of initial nanoliposome increased from 85 to 90 and 91 by adding the layers of WPC and pectin (Table 3).

### Assessment of Z-average, PdI and zeta potential of multilayer nanoliposomes containing PCSE

**Fig. 3:**
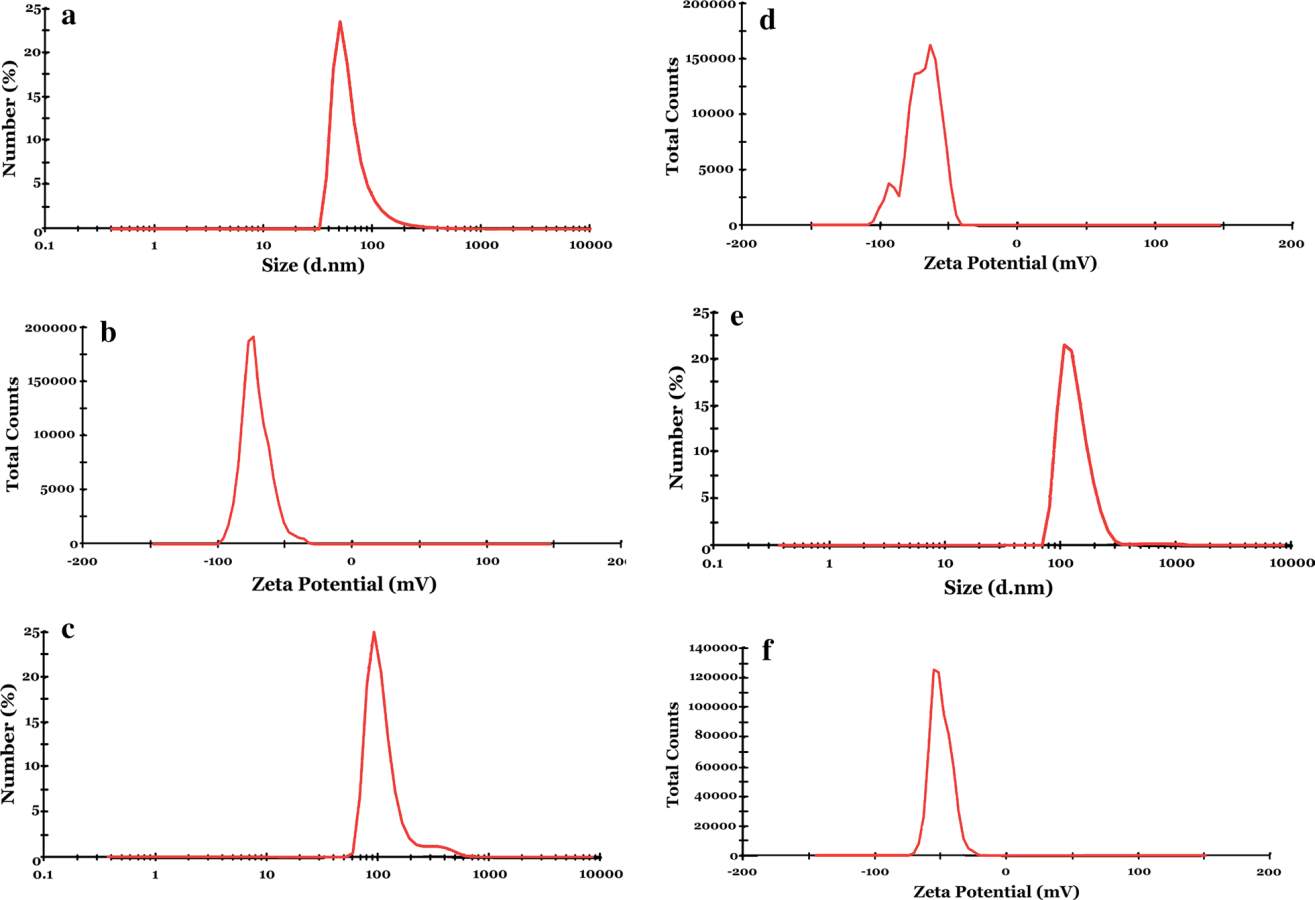
**a** Droplet size distribution of PCSEN; **b** droplet size distribution of PCSEN-W; **c** droplet size distribution of PCSEN-WP; **d** zeta potential of PCSEN; **e** zeta potential of PCSEN-W; **f** zeta potential of PCSEN-WP

The results of determining the z-average droplet size, PdI and zeta potential of PCSEN, PCSEN-W and PCSEN-WP are shown in Table 4 and Fig. 3.

**Table 4:** The Z-average droplet size, PdI and zeta potential of PCSEN, PCSEN-W and PCSEN-WP

Nanoliposome	Z-average (nm)	PdI	Zeta potential (mV)
PCSEN	282.5	0.424	− 56.9
PCSEN-W	481.7	0.531	− 53.5
PCSEN-WP	491.2	0.541	− 36.3

Particle size is the most important factor that can determine the success rate of microencapsulation, the compound loading into the coverage and the compound release. The particle size range in nanoscience depends on several different sources. IUPAC Association stated that the upper limit of nano-sized particles can be regarded as 500 nm in the case of considering some features such as transparency or opacity of solutions, passing through filters in the size of UF and/or preparing emulsions with high stability [39]. Solans and Soĺe [40] also categorized nanoemulsions as emulsions involving the size range 20–500 nm. The PCSEN, PCSEN-W and PCSEN-WP had z-average droplet size of 282.5, 481.7 and 491.2 nm, respectively (Table 4). According to our results, PCSEN, PCSEN-W and PCSEN-WP were within the range of nano emulsions that IUPAC was reported. The size distribution by number (%) of PCSEN, PCSEN-W and PCSEN-WP has been shown in Fig. 3a, d and e, respectively. It proved that there were many dispersed droplets with a size well below 500 nm. Compared to several studies conducted in the field of nanoliposomes, the samples produced in this study were within acceptable limits. The particle size distribution determines the amount of the produced nanoliposomes’ uniformity size. So that, whatever its amount is smaller the produced vesicles are more uniform in terms of size and consequently, a more favorable nanoliposome system will also be created.

The polydispersity index is defined as a dimensionless quantity of the broadness of the size distribution computed from the accumulation analysis, which ranges from 0 to 1 [41]. The polydispersity index value of 0.08 to 0.7 is the range, in which the distribution algorithms will operate in the best way. The results of the polydispersity index (PdI) indicated that particles had desirable status in terms of the size distribution, so that the particle size distribution for PCSEN, PCSEN-W and PCSEN-WP was respectively, 0.424, 0.531 and 0.541 (Table 4). Several different amounts have been reported for particle size distribution in various reports, which varies between less than 0.1 to greater than 0.7. Thus, the values obtained in the present study indicated a favorable situation for nanoemulsions.

Zeta potential is the surface charge of nanoparticles that affects on their physical state in liquids (e.g. stability, aggregation in a dispersion, absorption, etc.) and, thus, their interactions with biological systems [42]. The zeta potential value is between − 100 and + 100. Nanoparticles with a zeta potential less than − 25 and more than + 25 can produce stable nanoemulsion systems [43]. The zeta potential results of the samples also calculated (Table 4), which their results for PCSEN, PCSEN-W and PCSEN-WP were, respectively, − 56.9, − 53.5 and − 36.3, as shown in Fig. 3b, d and f, respectively. Therefore, zeta potential values of the prepared nanoliposomal systems proved that they were sustainable nanoliposomal systems. Martinez [44] used different methods to prepare nanoliposomes containing carvacrol. The particle size, particle size distribution and zeta potential of the produced samples were measured. As, the particle size, zeta potential, and particle size distribution were respectively in the range of 162 to 381 nm, − 40 to − 57 and 0.3 to 0.7. Their finding was consistent with the results of this study.

### Evaluation of antioxidant activity of the extract before and after coating against the oxidation process in soybean oil based on PV and TBA assays

The peroxide value assay characterizes primary reaction products of lipid oxidation, which can measure by their ability to release iodine from potassium iodide [45]. This index reveals the concentration of hydroperoxides which are unstable and they can be easily changed to alcohols, aldehydes, ketones and free fatty acids [46]. The TBA assay distinguishes the quantity of malondialdehyde (MDA) as a main secondary by-product of lipid oxidation in food and biological systems.

Tables 5 and 6 show the results of PV and TBA, respectively. As it is given in Tables, all treatments were delayed the oxidation of soybean oil in comparison to the control sample in all 20 days. In doing so, the PV in all samples was enhanced during the storage time. The increase in the amount of PV can be attributed to the formation of hydroperoxides [40]. The significant reduction rate of PV was observed in nanoliposomal forms of the extract than the control sample. Furthermore, the effect of concentration on the delaying of oxidation was a significant factor (Table 5). While concentration in a treatment was enhanced, its PV was decreased. Among all samples, PCSEN-WP and BHT were shown the highest inhibition of oxidation. In all days, the highest peroxide value was related to the control treatment. Also, PCSEN-WP sample at 300 ppm concentration had the highest performance in delaying the oil oxidation than other treatments. Overall, different samples related to their impact on lessening PVs are given from the highest to the lowest, as it follows:

**Table 5:** Peroxide values (meq/kg oil) of different treatments from PSCE during 20 days at 63 °C

Treatment	Day					
	0	4	8	12	16	20
Control	0.093 ± 0.00^Fa^	5.65 ± 0.13^Ea^	11.05 ± 0.30 ^Da^	13.92 ± 0.42^Ca^	20.15 ± 0.646^Ba^	28.45 ± 0.97^Aa^
BHT-100	0.065 ± 0.00^Fb^	1.35 ± 0.03^Eh^	4.40 ± 0.30^Dg^	7.85 ± 0.24^Ch^	10.79 ± 0.347^Bfg^	12.82 ± 0.438^Ae^
BHT-200	0.063 ± 0.00^Fb^	1.20 ± 0.03^Ei^	4.17 ± 0.11^Dg^	7.60 ± 0.41^Chi^	10.34 ± 0.332^Bgh^	12.24 ± 0.42^Aef^
PSCE-100	0.045 ± 0.00^Fg^	5.05 ± 0.11^Eb^	7.36 ± 0.20^Db^	9.56 ± 0.29^Cb^	13.28 ± 0.417^Bb^	16.45 ± 0.46^Ab^
PSCE-200	0.043 ± 0.00^Fh^	3.95 ± 0.09^Ec^	6.53 ± 0.30^Dc^	8.92 ± 0.20^Ccde^	12.42 ± 0.399^Bbc^	16.05 ± 0.55^Abc^
PSCE-300	0.005 ± 0.001 ^fg^	3.55 ± 0.09^Ed^	6.15 ± 0.27^Dcd^	8.66 ± 0.26^Cdef^	12.19 ± 0.392^Bbcd^	15.79 ± 0.54^Abc^
PSCEN-100	0.055 ± 0.01^Fcd^	2.53 ± 0.06^Ee^	5.82 ± 0.16^Dc^	9.33 ± 0.38^Cbc^	12.58 ± 0.404^Bbc^	15.96 ± 0.34^Abc^
PSCEN-200	0.056 ± 0.001^Fc^	1.95 ± 0.04^Eg^	5.17 ± 0.15^Def^	8.52 ± 0.36^Cef^	11.97 ± 0.385^Bcde^	15.60 ± 0.53^Abcd^
PSCEN-300	0.053 ± 0.00^Fde^	1.93 ± 0.04^Eg^	5.35 ± 0.15^Def^	8.42 ± 0.25^Cefg^	11.84 ± 0.48^Bdec^	15.08 ± 0.51^Acd^
PSCEN-W100	0.054 ± 0.05^Fcde^	3.55 ± 0.08^Ed^	6.05 ± 0.17^Dd^	9.29 ± 0.28^Cbc^	12.30 ± 0.295^Bbcd^	15.84 ± 0.54^Abc^
PSCEN-W200	0.053 ± 0.00^Fde^	1.95 ± 0.04^Eg^	5.40 ± 0.15^De^	8.40 ± 0.31^Cfg^	11.50 ± 0.37^Bde^	15.09 ± 0.42^Abc^
PSCEN-W300	0.055 ± 0.00^Fcd^	2.15 ± 0.05^Ef^	5.17 ± 0.15^Df^	7.93 ± 0.24^Cgh^	11.23 ± 0.36^Bef^	14.64 ± 0.50^Ad^
PSCEN-WP100	0.050 ± 0.00^Ff^	2.25 ± 0.05^Ef^	5.15 ± 0.16^Def^	7.09 ± 0.21^Cij^	9.06 ± 0.20^Bh^	12.85 ± 0.44^Ae^
PSCEN-WP200	0.052 ± 0.00^Fe^	2.15 ± 0.05^Ef^	4.25 ± 0.12^Dg^	6.80 ± 0.31^Cij^	8.92 ± 0.29^Bhi^	11.54 ± 0.19^Afg^
PSCEN-WP300	0.056 ± 0.00^Fc^	1.87 ± 0.04^Eg^	3.85 ± 0.11^Dh^	6.66 ± 0.20^Cj^	8.64 ± 0.18^Bi^	11.20 ± 0.38^Ag^

Differences between small letters show significant differences between different samples in each column (*p* ≤ 0.05)

Differences between capital letters indicate significant difference in each row (*p* ≤ 0.05)

**Table 6:** Thiobarbituric acid values (mg malonaldehyde/kg oil) of different samples of PCSE extracts, and BHT during 12 days at 63 °C

Treatment	Day					
	0	4	8	12	16	20
Control	0.033 ± 0.001^Da^	0.042 ± 0.002^Ca^	0.047 ± 0.002^Cab^	0.052 ± 0.003^Ca^	0.066 ± 0.001^Ba^	0.075 ± 0.006^Aa^
BHT-100	0.023 ± 0.003^Fb^	0.025 ± 0.001^Eh^	0.030 ± 0.003^Di^	0.034 ± 0.001^Ce^	0.038 ± 0.002^Bcd^	0.043 ± 0.004^Acde^
BHT-200	0.022 ± 0.001^Eb^	0.027 ± 0.004^Dgh^	0.029 ± 0.001^Di^	0.033 ± 0.002^Ce^	0.036 ± 0.001^Bde^	0.040 ± 0.002^Ade^
PSCE-100	0.006 ± 0.000^Ef^	0.032 ± 0.001^Dbc^	0.037 ± 0.003^Ce^	0.040 ± 0.002^Bcd^	0.045 ± 0.003^Ab^	0.043 ± 0.001^ABbcde^
PSCE-200	0.007 ± 0.000^Ee^	0.029 ± 0.001^DFg^	0.034 ± 0.001^Cg^	0.036 ± 0.002^BCde^	0.043 ± 0.002^Abc^	0.039 ± 0.005^ABe^
PSCE-300	0.005 ± 0.003^Dfg^	0.032 ± 0.002^Cbc^	0.042 ± 0.001^Abcd^	0.034 ± 0.003^Be^	0.041 ± 0.004^Abc^	0.043 ± 0.002^Ac^
PSCEN-100	0.007 ± 0.000^Ee^	0.031 ± 0.001^Dce^	0.033 ± 0.000^Ch^	0.040 ± 0.002^ABcd^	0.043 ± 0.002^Ab^	0.037 ± 0.001^Be^
PSCEN-200	0.005 ± 0.000^Fg^	0.032 ± 0.002^CDbc^	0.035 ± 0.002^BCfg^	0.039 ± 0.002^Ad^	0.033 ± 0.000^Df^	0.029 ± 0.002^Ef^
PSCEN-300	0.008 ± 0.002^Ecd^	0.034 ± 0.004^Bb^	0.031 ± 0.002^Chi^	0.037 ± 0.000^ABde^	0.041 ± 0.001^Abc^	0.024 ± 0.003^Dg^
PSCEN-W100	0.008 ± 0.000^Ede^	0.03 ± 0.002^Dcef^	0.043 ± 0.003^Bbc^	0.037 ± 0.001^Cd^	0.048 ± 0.004^ABb^	0.048 ± 0.003^Ab^
PSCEN-W200	0.007 ± 0.001^Ede^	0.028 ± 0.000^Dfgh^	0.049 ± 0.000^Aa^	0.036 ± 0.004^Cde^	0.045 ± 0.002^Bb^	0.048 ± 0.005^ABCbcd^
PSCEN-W300	0.010 ± 0.000^Dcd^	0.027 ± 0.000^Ch^	0.041 ± 0.002^Bcd^	0.043 ± 0.002^ABbc^	0.043 ± 0.003^ABb^	0.045 ± 0.002^Abc^
PSCEN-WP100	0.005 ± 0.003^Fg^	0.028 ± 0.001^Efgh^	0.040 ± 0.000^Cd^	0.047 ± 0.002^Aab^	0.033 ± 0.002^Def^	0.042 ± 0.003^Bde^
PSCEN-WP200	0.005 ± 0.001^Dfg^	0.027 ± 0.001^Cgh^	0.036 ± 0.000^Bf^	0.045 ± 0.003^Aabc^	0.041 ± 0.003^Abc^	0.044 ± 0.002^Ac^
PSCEN-WP300	0.006 ± 0.000^Df^	0.029 ± 0.000^CF^	0.033 ± 0.001^Bghi^	0.039 ± 0.004^Ad^	0.033 ± 0.004^Bf^	0.037 ± 0.001^Ae^

Differences between small letters show significant differences between different samples in each column (*p* ≤ 0.05)

Differences between capital letters indicate significant difference in each row (*p* ≤ 0.05)

Control > PCSE-100 > PCSE-200 > PCSEN-100 > PCSEN-W100 > PCSE -300 > PCSEN-200 > PCSEN-W200 > PCSEN-300 > PCSEN-W300 > PCSEN-WP100 > BHT-100 > BHT-200 > PCSEN-WP200 > PCSEN-WP300.

According to the results shown in Table 6, thiobarbituric acid values of different samples were at the minimum level. The TBA values were increased (*p *< 0.05) for all treatments during the 20-day testing period (Table 6). In this process, aldehydes might be oxidized to carboxylic acids; consequently, the amount of TBA will be reduced. In each of the 20 days, the highest and lowest of TBA values were belonged to the control and PCSEN-300 samples, respectively. Because of the presence of antioxidant agents, it seems that the extracts in both free and nanoliposomal forms had a negative effect on the TBA value so that they had the lowest TBA in comparison to BHT-100 and BHT-200 (Table 6) even in the least concentration (100 ppm).

These findings were in agreement with the results of Mohammadi et al. [33]. Their results exhibited that nano-encapsulation of olive leaf extract could control the peroxide value better than simple olive leaf extract during storage of soybean oil. In the other research, Chatterjee and Bhattacharjee [47] evaluated the effects of encapsulated and un-encapsulated eugenol-rich clove extracts on soybean oil oxidation. Their results proved that there was no significant difference between antioxidant activities of various forms of the extracts in soybean oil.

## Conclusion

The results showed that methanolic extract of PCS had the highest amount of total phenol and free radical scavenging among its various extracts. The encapsulation structures based on PCSE-loaded nanoliposomes prepared by using six different formulations containing various proportions of the oil, lecithin and the extract. The findings of creaming index and microencapsulation efficiency indicated that the formulation containing 30% the oil, 5% lecithin and 2% the extract was led to produce the best nanoliposomal structure (PCSEN). Then, single and double layer nanoliposomal structures produced by using the WPC and pectin (PCSEN-W and PCSEN-WP, respectively). The results of determining the size of nanoliposomal particles showed that z-average size of them ranged from 282.5 to 491.2 nm. The PdI of nanoliposomal systems were 0.424–0.541. The zeta potential of PCSEN, PCSEN-W and PCSEN-WP were, respectively − 56.9, − 53.5 and − 36.3 and they indicated that the nanoliposomal systems of PCS were stable. Also, the results of peroxide and thiobarbituric acid values of the extracts in free and nanoliposomal forms exhibited that they had very good antioxidant activity against the oxidation process in soybean oil.

## Data Availability

All data and material analyzed or generated during this investigation are included in this published article.

## Abbreviations

PCSE: *Persian* clover sprout
WPC: whey protein concentrate
PCSEN: nanoliposome containing PCSE
PCSEN-W: PCSE-loaded functionalized nanoliposome produced using WPC
PCSEN-WP: PCSE-loaded functionalized nanoliposome produced using WPC and pectin
PV: peroxide value
TBA: thiobarbituric acid
